# Suppression of GCH1 Sensitizes Ovarian Cancer and Breast Cancer to PARP Inhibitor

**DOI:** 10.1155/2023/1453739

**Published:** 2023-02-06

**Authors:** Siyuan Wang, Yu Xia, Pu Huang, Cheng Xu, Yiyu Qian, Tian Fang, Qinglei Gao

**Affiliations:** ^1^Cancer Biology Research Center (Key Laboratory of Chinese Ministry of Education), Tongji Hospital Tongji Medical College Huazhong University of Science and Technology, 1095 Jiefang Ave, Wuhan 430030, China; ^2^Department of Gynecology and Obstetrics, Tongji Hospital Tongji Medical College Huazhong University of Science and Technology, Wuhan 430030, China

## Abstract

**Background:**

Breast and ovarian cancers are common malignancies among women, contributing to a significant disease burden, and are characterized by a high level of genomic instability, owing to the failure of homologous recombination repair (HRR). Pharmacological inhibition of poly(ADP-ribose) polymerase (PARP) could elicit the synthetic lethal effect of tumor cells in patients with homologous recombination deficiency, ultimately achieving a favorable clinical benefit. However, primary and acquired resistance remain the greatest hurdle, limiting the efficacy of PARP inhibitors; thus, strategies conferring or augmenting tumor cell sensitivity to PARP inhibitors are urgently required.

**Methods:**

Our RNA-seq data of niraparib-treated and -untreated tumor cells were analyzed by R language. Gene Set Enrichment Analysis (GSEA) was applied to assess the biological functions of GTP cyclohydrolase 1 (GCH1). Quantitative real-time PCR, Western blotting, and immunofluorescence were applied to confirm the upregulation of GCH1 upon niraparib treatment at transcriptional and translational levels. Immunohistochemistry of patient-derived xenograft (PDX)-derived tissue sections further validated that niraparib increased GCH1 expression. Tumor cell apoptosis was detected by flow cytometry, while the superiority of the combination strategy was confirmed in the PDX model.

**Results:**

The expression of GCH1 was aberrantly enriched in breast and ovarian cancers and increased after niraparib treatment via JAK-STAT signaling. GCH1 was also demonstrated to be associated with the HRR pathway. Subsequently, the enhancement of the tumor-killing effect of PARP inhibitors induced by GCH1 suppression using siRNA and GCH1 inhibitor was validated by flow cytometry in vitro. Finally, using the PDX model, we further demonstrated that GCH1 inhibitors markedly potentiated PARP inhibitors' antitumor efficacy in vivo.

**Conclusion:**

Our results illustrated that PARP inhibitors promote GCH1 expression via the JAK-STAT pathway. We also elucidated the potential relationship between GCH1 and the homologous recombination repair pathway and proposed a combination regimen of GCH1 suppression with PARP inhibitors in breast and ovarian cancers.

## 1. Introduction

Ovarian and breast cancers, regarded as the two major malignancies threatening women's health [1], have limited therapeutic options other than surgical resection combined with adjuvant chemotherapy and face more daunting challenges that the majority of patients will experience chemotherapy resistance and relapse [2, 3]. With more targeted therapies springing up, the management of ovarian and breast cancer has been revolutionized and stepped into a new era.

Poly(ADP-ribose) polymerase (PARP), first known for its catalytic enzyme activity in the process of poly(ADP- ribosyl)ation, was subsequently verified to play crucial roles in a series of cellular processes such as timely and accurate DNA repair and cell death [4, 5]. Pharmacological inhibitors of PARP have shown promising therapeutic potential in various cancers. Results from a phase 3 trial PRIMA/ENGOT-OV26/GOG-3012 indicated a statistically significant extension of progression-free survival (PFS) in patients who received niraparib compared with placebo among newly diagnosed advanced ovarian cancer patients [6]. Olaparib, the first PARP inhibitor, was demonstrated to provide a substantial PFS benefit for patients with BRCA1/2 mutations and HER2-negative breast cancer, reducing the risk of disease progression or death by 42% in comparison with standard therapy [7]. As of 2020, PARP inhibitors have gained approval in ovarian cancer, breast cancer, prostate cancer, and pancreatic adenocarcinoma [6–9], suggesting that PARP inhibitors might have the ability to shift the treatment paradigm for these cancers. However, the population sensitive to PARP inhibitors is mostly limited to patients with homologous recombination deficiency (HRD) due to their synthetic lethal effects. Therefore, an improved understanding of the biology of cancer will facilitate the development of rational strategies to increase the sensitivity to PARP inhibitors and expand the applicable population.

Whilst PARP inhibitors initially present favorable tumor-killing effects, most patients would experience acquired drug resistance over time, triggering tumor progression. Resistance to PARP inhibitors is now recognized to be acquired primarily through the following mechanisms: restoration of homologous recombination function, stability and protection of replication forks, enhancement of drug efflux transporters, and PARP-related mechanisms [10]. In response to these possible resistance mechanisms, many studies have focused on combination regimens in an attempt to combat the emergence of acquired resistance with the aim of amplifying the antitumor effects of PARP inhibitors. PI3K-AKT pathway inhibitors, which have been reported to impair homologous recombination repair (HRR), render the tumor cells more sensitive to PARP inhibitors [11–14]. Suppression of the NAD^+^ salvage pathway using the NAMPT inhibitor FK866 led to inhibition of PARylation, resulting in potent enhancement of olaparib cytotoxicity in triple negative breast cancer [15]. Furthermore, the field of immune checkpoints has been remarkably vigorous in the last decades, and its combination with PARP inhibitors is also an area of active investigation. The benefits of PARP inhibitors in combination with anti-PD-1/anti-PD-L1 can manifest themselves via increased objective response rates in patients [16, 17], probably related to the activation of cGAS-STING signaling and increased expression of PD-L1 [18, 19]. Indeed, metabolic reprogramming is also closely associated with the sensitivity of PARP inhibitors. Suppression of an important glycolytic enzyme, phosphoglycerate mutase 1 (PGAM1), sensitized BRCA1/2-proficient breast cancer to PARP inhibitors by impairing homologous recombination repair [20]. These preclinical and clinical data given above have sparked a rapidly growing interest in the combination strategies of PARP inhibitors.

In this study, we have uncovered that niraparib could upregulate the expression of GTP cyclohydrolase 1 (GCH1), a vital rate-limiting enzyme for the cofactor of nitric oxide synthases (NOSs) tetrahydrobiopterin (BH4) synthesis and an important participant in many chronic diseases, even malignant tumors, through JAK-STAT signaling [21–24]. Moreover, we clarified that the suppression of GCH1 could sensitize tumor cells to the PARP inhibitor, in order to better ameliorate clinical management of ovarian and breast cancers.

## 2. Materials and Methods

### 2.1. RNA Extraction and Library Construction

SKOV3 cells (human ovarian cancer cell line obtained from American Type Culture Collection, ATCC) were seeded in T75 culture flask at a suitable density and cultured overnight to adhere. Cells were treated with 10 *μ*M niraparib (ZL-2306, Zai Lab, China) or 0.2% DMSO for 72 hours prior to collection. Each group contained three parallel replicates. The total RNA was quantified using a Nano Drop and Agilent 2100 bioanalyzer (Thermo Fisher Scientific, MA, USA) after being extracted using Trizol (Invitrogen, Carlsbad, CA, USA) according to the manufacturer's instructions. Purified mRNA was used for cDNA synthesis, followed by the construction of the final library which was further sequenced on the MGISEQ 2000 platform (BGI Corporation, Shenzhen, China).

### 2.2. Analysis of Differentially Expressed Genes (DEGs)

A list of 2,752 metabolism-related genes (MRGs) was obtained from a previous study [25]. The differentially expressed MRGs between the niraparib-treated and -untreated groups were identified using the DESeq2 R package (|log2(fold change)| > 1 and adjusted *P* value < 0.05) [26]. Heatmaps and volcano plots were visualized via ggplot2, ggdendro, and EnhancedVolcano R packages [27, 28].

### 2.3. Cell Culture and Transfection

Human breast cancer cell line MDA-MB-231 and HCC38 and ovarian cancer cell line A2780 and OVCAR8 were obtained from ATCC and cultured in DMEM or RPMI 1640 medium containing 10% FBS and 1% penicillin-streptomycin solution (G4003, Servicebio, China). GCH1 siRNAs and control siRNA were purchased from RiboBio Co. Ltd. (Guangzhou, China). The transfection of siRNA was performed according to the manufacturer's instruction and knockdown efficacy was examined by real-time PCR after 48 hours.

### 2.4. Isolation of RNA and Quantitative Real-Time PCR

The total RNA was extracted using the FastPure Cell/Tissue Total RNA Isolation Kit (RC101, Vazyme), while cDNA was further synthesized using HiScript II Q RT SuperMix for qPCR (R223-01, Vazyme). The ChamQ Universal SYBR qPCR Master Mix (Q711-02, Vazyme) was applied to quantitative real-time PCR. All primers used were as follows:

  GCH1-F GCCATGCAGTTCTTCACCAAGG
  GCH1-R ATGGAACCAAGTGATGCTCACAC
  GAPDH-FTGCACCACCAACTGCTTAGC
  GAPDH-RGGCATGGACTGTGGTCATGAG

### 2.5. Western Blotting

For in vitro cell line samples, optimized density of tumor cells was seeded prior to treatment. After being treated for the indicated time and concentration, cells were rinsed with ice-cold PBS and lysed in RIPA buffer with proteases and a phosphatase inhibitor (78440, Thermo Fisher Scientific). Denatured cell extracts were subjected to SDS-PAGE gel electrophoresis before being transferred to PVDF membranes. Blots were blocked and incubated with anti-GCH1 antibodies (ab236387, Abcam) or anti-GAPDH (GB11002, Servicebio, China) for 12–16 hours at 4°C, followed by incubation with appropriate horseradish peroxidase-conjugated antibodies at room temperature for 1 hour. Immunoblotting was detected by the ECL chemiluminescence system (Bio-Rad).

### 2.6. Immunofluorescence

Cells were seeded in 12-well plates and treated with niraparib for the indicated time. After being washed twice with PBS, cells were fixed with 4% PFA and permeabilized with 0.5% Triton X-100. Cells were blocked with 5% BSA prior to staining with primary antibodies against GCH1 (A10616, Abclonal), *γ*-H2AX (GB111841, Servicebio, China), or RAD51 (GB11572, Servicebio, China) overnight, followed by incubation with appropriate secondary antibodies. DAPI (G1012, Servicebio, China) was used to stain nuclei for 8 min. Images were acquired on fluorescent microscopy.

### 2.7. Immunohistochemical Staining of Tissue Sections

After being fixed with formalin and embedded with paraffin, tumor tissues were sliced into slides. Paraffin sections were heated at 65–68°C for 2 hours before being deparaffinized in a dewaxing agent and rehydrated sequentially. Antigen retrieval was performed using water bath heating and endogenous peroxidase was consumed in 3% H_2_O_2_. After blocking with 5% BSA for 30 min at 37°C, sections were incubated with primary antibodies against GCH1 (ab236387, Abcam) overnight at 4°C and secondary antibodies at room temperature for 1 hour. DAB was applied for color development. Sections were counterstained with hematoxylin, dehydrated by graded ethanol, and coverslipped before being visualized with an Olympus microscope. The IHC score was assessed based on staining intensity score (0, no staining; 1, weak staining; 2, moderate staining; and 3, intense staining) and the proportion score (1, 0–25% of the tumor cells were stained; 2, 26–50%; 3, 51–75%; and 4, more than 75% of the tumor cells were stained). IHC score = staining intensity score × proportion score.

### 2.8. Data Source and Preparation

RNA expression profiles and clinical information from breast invasive carcinoma (BRCA) project (include 292 normal and 1,099 cancerous tissues, and a total of 112 paired normal and tumor tissues) and ovarian serous cystadenocarcinoma (OV) project (include 88 normal and 427 cancerous tissues) were obtained from the Cancer Genome Atlas (TCGA) database. All raw RNA-seq data were transformed from FPKM format into transcripts per million reads (TPM) format for further studies.

### 2.9. Function Analysis of GCH1-Associated DEGs

As for the identification of DEGs between high- and low-GCH1 expression groups, the threshold was determined according to the parameters, adjusted *P* value < 0.05, and the absolute log-fold change larger than 1.5. The identified DEGs were then processed for the Gene Set Enrichment Analysis (GSEA) using the R package clusterProfiler (3.8.0) and the thresholds were set as adjusted *P* value <0.05 and FDR < 0.25. Gene Ontology (GO) and Kyoto Encyclopedia of Genes and Genomes (KEGG) were conducted to explore the potential function of GCH1-associated DEGs. The protein-protein interaction (PPI) network was constructed according to the online STRING database (https://string-db.org/). The CHIP-seq data were collected from Cistrome Data Browser (http://cistrome.org/db).

### 2.10. Clinical Significance of GCH1

The relationship between the expression level of GCH1 and clinicopathological characteristics was assessed with the Wilcoxon signed-rank sum test and logistic regression. All the statistical analyses given above were performed in R (v3.6.3), and *P* values <0.05 were considered significant.

### 2.11. Cell Apoptosis Detection

The MDA-MB-231 cells were incubated with 10 *μ*M niraparib for indicated times after transfection by siRNAs for 48 hours followed by apoptosis quantification. A2780 and MDA-MB-231 cells were treated with 0.2% DMSO, 5 *μ*M GCH1 inhibitor DAHP (S3688, Selleck), 10 *μ*M niraparib, or a combination for 24, 48, or 72 hours before apoptosis was assessed. Tumor cell apoptosis was measured using Apoptosis Detection Kit I (556547, BD Biosciences) according to the instructions.

### 2.12. Colony Formation Assay

500 MDA-MB-231 or A2780 cells were seeded in each well of 6-well plates and allowed to adhere for 4 hours prior to treating with 0.2% DMSO, 5 *μ*M DAHP, 10 *μ*M niraparib, or a combination for 5–10 days. Cells were then fixed for 30 min and stained with crystal violet for 1 hour and counted.

### 2.13. Cell Viability Assay

Cells were seeded in a 96-well plate and treated with 10 *μ*M niraparib for indicated times and wells were replaced with fresh medium containing CCK8 (A311-01, Vazyme). The cell viability was measured according to the protocols.

### 2.14. Establishment of the Patient-Derived Xenograft (PDX) Model

Fresh tumor tissue obtained from surgery was preserved in saline solution and immediately transported to the laboratory on ice within 4 hours. The tumor tissues were trimmed and cut into small fragments. 6–8-week-old female NOD-Prkdc^scid^Il2rg^em1^/Smoc micewere preanesthetized with 1%–1.5% isoflurane (R510-22-10, RWD Life Cycle) and the tissue pieces were implanted subcutaneously on the dorsum of the mice to produce the first generation xenografts. The size of the tumor was measured every 2 days thereafter. When the maximum tumor diameter was about 15–20 mm, the micewere euthanized and the tumor tissue was transplanted into new NOD-Prkdc^scid^Il2rg^em1^/Smoc mice as the next generation models.

### 2.15. In Vivo Animal Studies

The animal study was approved by the Animal Care and Use Committee of Tongji Hospital, Tongji Medical College, Huazhong University of Science and Technology. Twenty 6–8 weeks-old female NOD-Prkdc^scid^Il2rg^em1^/Smoc mice were anesthetized with 1%–1.5% isoflurane (R510-22-10, RWD Life science, Shenzhen, China). Subsequently, patient-derived xenografts (PDXs) were engrafted subcutaneously on the dorsum. After inoculation, mice weights and tumor sizes were measured twice weekly. When the tumors reached 50–100 mm^3^, mice were randomized into four subgroups. Niraparib was dosed (20 mg/kg, intragastric administration) once daily for 5 days per week while DAHP was dosed (60 mg/ml, intraperitoneal administration) once daily. Tumor volume based on caliper measurements will be calculated using the modified ellipsoidal formula: tumor volume = 1/2 length × width^2^. At experimental endpoints, the mice will be sacrificed after anesthesia. Tissue samples were taken for routine hematoxylin and eosin (HE) staining and immunohistochemistry.

### 2.16. Statistical Analysis

GraphPad Prism 9 was employed in statistical analysis to graph our data. Student's *t*-test was used for comparison between two groups, and one-way ANOVA analysis was applied to the comparison of more than two groups. Time course data were analyzed by two-way ANOVA. All results are represented as the mean ± SD. *P* < 0.05 was considered statistically significant and denoted as follows: ^*∗*^*P* < 0.05, ^*∗∗*^*P* < 0.01, and ^*∗∗∗*^*P* < 0.001.

## 3. Results 

### 3.1. PARP Inhibitor Promotes the Transcription and Translation of GCH1 in Breast and Ovarian Cancers

**Figure 1:**
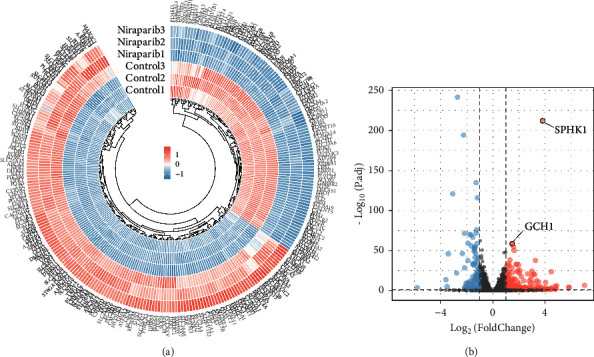
GCH1 was upregulated in niraparib-treated groups. (a) Heatmap and (b) volcano plots of differentially expressed metabolism-related genes under niraparib administration.

To clarify the alterations of tumor cells after PAPR inhibitors administration, providing clues to the mechanism of drug resistance, niraparib-treated and -untreated SKOV3 cells were subjected to RNA-seq and the DEGs were identified by Wilcoxon rank-sum test (Supplementary Table 1). We matched these DEGs with a list of 2,752 MRGs that encoded almost all known human metabolic enzymes and transporters. Considering the cutoff criteria (adjust *P* value < 0.05 and log∣FC∣ > 1.0), a total of 276 differentially expressed MRGs containing 172 upregulated and 104 downregulated genes were extracted (Figure 1(a)). Representative DEGs were also illustrated in volcano plot (Figure 1(b)). Among the upregulated DEGs in the niraparib-treated group, SPHK1 was the most significantly changed gene, but the difference in expression between the tumor and the corresponding normal tissue was inconspicuous in breast and ovarian cancers according to the TCGA and GTEx databases (Supplementary Figures 1(a) and 1(b)). So, we focused on GCH1, which had a relatively significant difference.

**Figure 2:**
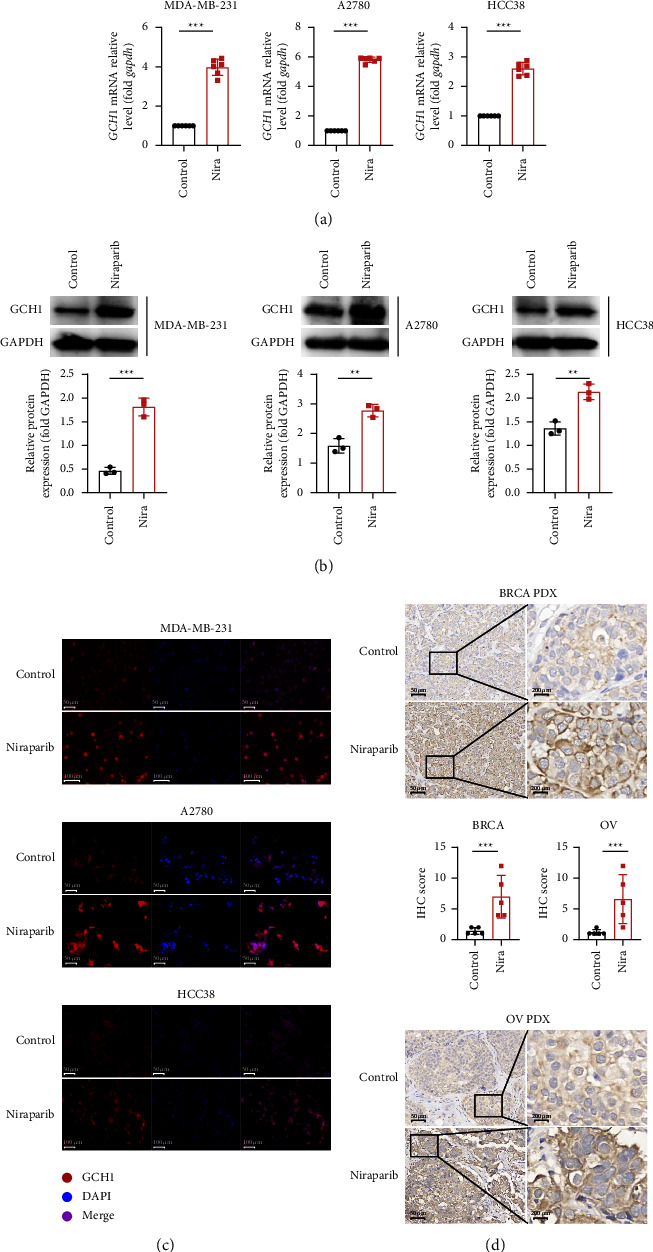
GCH1 was increased upon niraparib treatment at transcriptional and translational levels. GCH1 transcription was analyzed by (a) qRT-PCR in MDA-MB-231, A2780 and HCC38 cell lines after treated with 10 *μ*M niraparib for 48 h. The expression of GCH1 was measured by (b) western blot in MDA-MB-231, A2780 and HCC38 cell lines after treated with 10 *μ*M niraparib for 72 h. (c) Representative immunofluorescence images of GCH1 expression in control group and cells treated with 10 *μ*M niraparib for 72 h. (d) Representative images of IHC staining of GCH1 in human ovarian and breast cancer tissues derived from PDX models are shown, and GCH1 expression was quantified according to the IHC score.

Consistent with the RNA-seq results, GCH1 mRNA levels were increased in multiple ovarian and breast cancer cell lines in response to niraparib treatment (Figure 2(a)). The niraparib-induced additional expression of GCH1 was validated by immunoblotting (Figure 2(b)). Likewise, immunofluorescence results confirmed that GCH1 was enriched in the presence of niraparib (Figure 2(c)). We further performed histochemical staining of clinical specimens derived from PDX models of breast and ovarian cancer and similarly verified that GCH1 expression was substantially higher in the niraparib-treated group compared with the control group (Figure 2(d)). In summary, the transcription and translation of GCH1 was remarkably elevated upon PARP inhibitor treatment.

### 3.2. Clinical Implications of GCH1 in Breast and Ovarian Cancers

**Figure 3:**
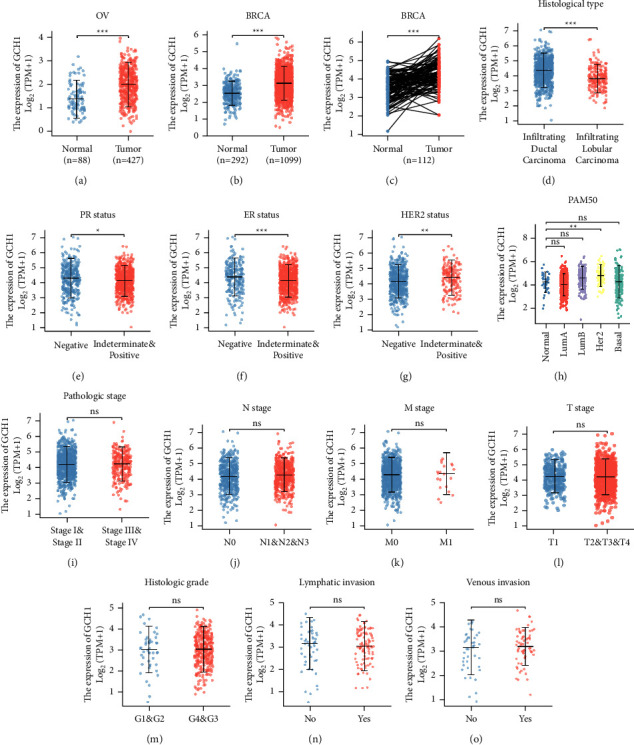
Association between GCH1 expression and the clinicopathological characteristics. GCH1 expression is higher in (a) ovarian tumor and (b) breast tumor in the TCGA and GTEx database. (c) Increased expression of GCH1 in breast cancerous tissue compared with paired normal tissue. GCH1 is correlated with (d) histological type, (e) PR status, (f) ER status, (g) HER2 status, and (h) PAM50 subtype classification in BRCA. GCH1 was not significantly correlated with (i) pathologic stage, (j) *N* stage, (k) *M* stage, and (l) *T* stage in breast cancer cohort. (m) Histologic stage, (n) lymphatic invasion, and (o) venous invasion were not significantly correlated with GCH1 expression in ovarian cancer cohort.

Given the limited research on the expression and function of GCH1 in breast and ovarian cancers, we initially explored GCH1 expression and its correlation with clinicopathological characteristics. Based on the TCGA and GTEx databases, we analyzed the expression of GCH1 mRNA. The results demonstrated that GCH1 expression was much higher in breast and ovarian cancers (Figures 3(a) and 3(b)). In addition, a similar result was obtained in breast cancer tissues and paired normal breast tissue (Figure 3(c)). The association between GCH1 expression and the clinical characteristics of patients with breast and ovarian cancers are summarized in Supplementary Tables 2 and 3. In breast cancer, high expression of GCH1 was significantly correlated with histological type, PR status, ER status, HER2 status, and Prediction Analysis of Microarray 50 (PAM50) subtype classification (Figures 3(d)–3(h)). However, breast cancer patients with different pathologic stages, *T* stages, *N* stages, and *M* stages shared comparable GCH1 expression levels (Figures 3(i)–3(l)). As for ovarian cancer, the relationship between GCH1 and clinicopathology was not remarkable. GCH1 expression had no distinct correlation with histologic grade, lymphatic invasion and venous invasion in ovarian cancer patients (Figures 3(m)–3(o)).

Furthermore, logistic regression analysis revealed the statistically significant association between GCH1 high expression and histological type, PR status, ER status, and HER2 status in breast cancer (Supplementary Table 4). A prominent correlation of GCH1 expression with Federation International of Gynecology and Obstetrics (FIGO) stage was observed in ovarian cancer as well (Supplementary Table 5).

### 3.3. PARP Inhibitor Induces the Accumulation of GCH1 through the JAK-STAT Pathway

**Figure 4:**
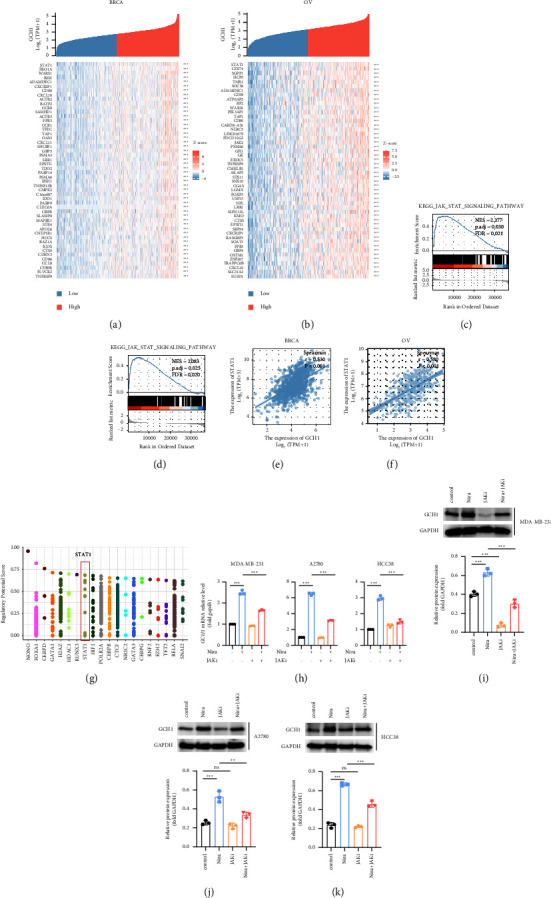
Identification of GCH1-associated DEGs and the potential transcription factor STAT1 of GCH1. Heat map showing the top 50 DEGs between high- and low-GCH1 expression groups in (a) BRCA and (b) OV. GSEA showing the enrichment of the JAK-STAT pathway in GCH1-high expression (c) breast and (d) ovarian cancer. Shown are correlation of GCH1 and STAT1 expressions in (e) BRCA and (f) OV cohorts, respectively. (g) Cistrome Data Browser indicated STAT1 regulated GCH1 transcription directly. (h) GCH1 mRNA were evaluated by qRT-PCR in MDA-MB-231, A2780, and HCC38 cells when treated with 10 *μ*M niraparib and/or 50 *μ*M JAK inhibitor for 48 h. (i–k) The protein level of GCH1 were determined by Western blot in MDA-MB-231, A2780, and HCC38 cells after treated with 10 *μ*M niraparib and/or 50 *μ*M JAK inhibitor for 72 h.

To further elucidate the potential mechanisms of GCH1 regulation, all the cases were divided into two groups and compared based on the median GCH1 expression in tumors. According to the threshold of |log2(FC)| > 1.5 and adjusted *P* value < 0.05, a total of 778 genes, covering 227 upregulated genes and 551 downregulated genes, were recognized as DEGs in breast cancer. While in ovarian cancer, 324 genes (including 252 increased and 72 decreased genes) were identified as statistically significant between the two cohorts. The top 50 genes positively correlated with GCH1 were illustrated by heatmaps (Figures 4(a) and 4(b)), and the top 50 DEGs negatively associated with GCH1 were shown in Supplementary Figures 2(a) and 2(b). Surprisingly, STAT1 ranked first in both breast cancer and ovarian cancer, implying that there might be a strong correlation between STAT1 and GCH1. The Gene Set Enrichment Analysis (GSEA) of all DEGs also showed significant enrichment of the JAK-STAT signaling pathway (Figures 4(c) and 4(d)). Further, the correlations of GCH1 and STAT1 were directly assessed. Given the Spearman correlation coefficients were 0.53 and 0.58, GCH1 and STAT1 were well-correlated in breast cancer (Figure 4(e)) and ovarian cancer (Figure 4(f)). Finally, we obtained CHIP-seq data from the Cistrome Data Browser showing that STAT1 could bind to the promoter region of GCH1 to play a regulatory role (Figure 4(g)). To validate the involvement of the JAK-STAT pathway in the regulation of GCH1, MDA-MB-231, A2780, and HCC38 cancer cell lines were subjected to JAK-STAT inhibitor ruxolitinib or/and niraparib. Consistent with the abovementioned findings, the JAK-STAT inhibitor alone exhibited a modest suppressive impact on GCH1 expression. In addition, niraparib has been reported to activate the JAK-STAT signaling pathway [29]. Our results revealed that inactivation of JAK-STAT signaling resulted in the rollback of niraparib-induced upregulation of GCH1, indicating that niraparib might modulate GCH1 via the JAK-STAT pathway (Figures 4(h)–4(k)).

### 3.4. GCH1 Correlates with the Homologous Recombination Repair (HRR) Pathway

**Figure 5:**
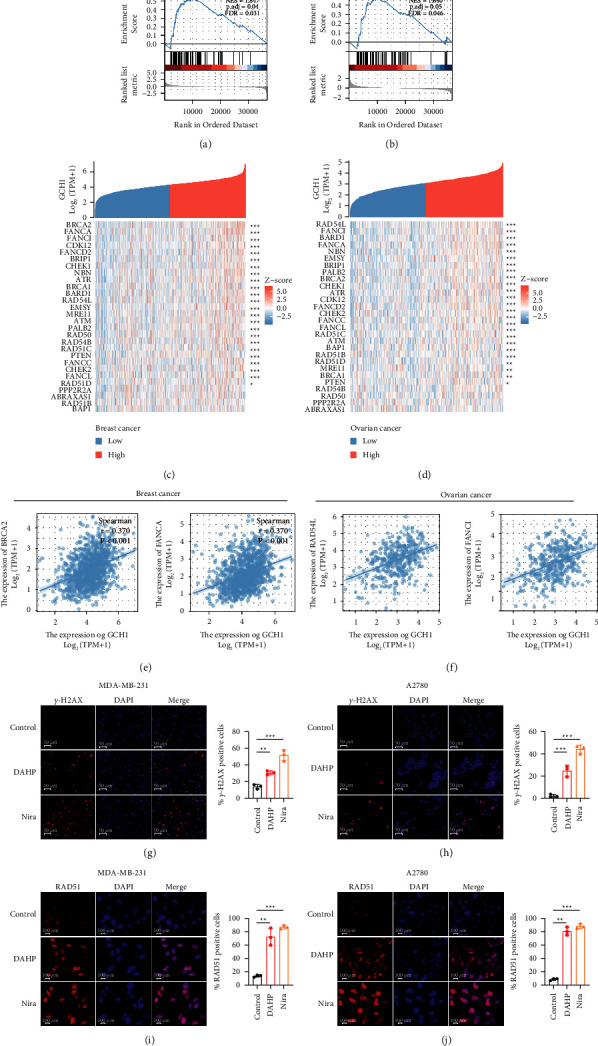
GCH1 was associated with homologous recombination repair (HRR) pathway. GSEA analysis of differentially expressed genes between high- and low-GCH1 expression groups showing the enrichment of homologous recombination repair (HRR) pathway in (a) breast cancer and (b) ovarian cancer. (c-d) Shown are expression profiles of 28 HRR-related genes in high- and low-GCH1 groups, and data are presented by heatmaps. Correlation diagrams showing the association between GCH1 expression and HRR-related genes, including FANCA and BRCA2 in (e) breast cancer and RAD54L and FANCI in (f) ovarian cancer. Representative immunofluorescence images and quantification of *γ*-H2AX foci and Rad51 foci formation in control and 5 mM DAHP-treated groups of (g, i) MDA-MB-231 cells and (h, j) A2780 cells. 10 *μ*M niraparib was used as positive control and all the drugs were administered for 24 hours.

We observed that the core molecules of HRR cluster were mainly enriched in the GCH1-high expression group (*P*=0.04) in breast cancer (Figure 5(a)). Although, there was no statistical significance between the two groups in ovarian cancer (*P*=0.05), the overall enrichment trend was similar to that of breast cancer (Figure 5(b)). Further, we analyzed the association between GCH1 and 28 genes highly related to the HRR pathway (Figures 5(c) and 5(d)) and Spearman's correlation coefficients greater than 0.3 were considered to indicate a certain correlation. In breast cancer, GCH1 was significantly related to BRCA2, FANCA, FANCI, CDK12, FANCD2, BRIP1, and CHEK1 (Figure 5(e), Supplementary Figures 3(a)–3(e)), while in ovarian cancer, GCH1 was positively associated with 11 genes, including RAD54L, FANCI, BARD1, FANCA, NBN, EMSY, BRIP1, PALB2, BRCA2, CHEK1, and ATR (Figure 5(f), Supplementary Figures 3(f)–3(n). Thus, we treated ovarian and breast cancer cell lines with GCH1 inhibitor DAHP and analyzed *γ*-H2AX foci formation. The administration of DAHP resulted in considerably higher DNA damage, as shown by the increased number of *γ*-H2AX-positive cells in the DAHP group compared to the control group (Figures 5(g) and 5(h)), indicating that GCH1 was involved in the DNA repair process. Moreover, the exposure to DAHP induced formation of Rad51 foci in the cell nucleus (Figures 5(i) and 5(j)). Taken together, GCH1 was demonstrated to be associated with HRR.

### 3.5. Synergistic Effect of GCH1 Inhibition and PARP Inhibitor In Vitro

**Figure 6:**
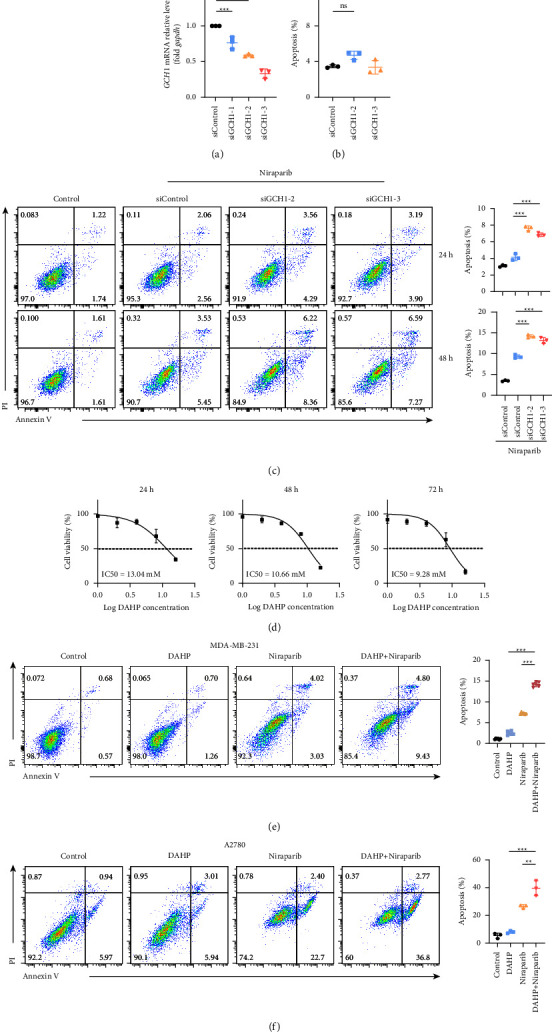
Inhibition of GCH1 potentiated PARP inhibitor therapy for cancers. (a) Quantitative real-time PCR assay of GCH1 in MDA-MB-231 cells 48 hours after transfection with indicated siRNAs. (b) Apoptosis of MDA-MB-231 cells was measured via flow cytometry after 48 hours of siRNA transfection. (c) MDA-MB-231 cells were treated with 10 *μ*M niraparib for indicated times after transfection with siRNAs and assessed by flow cytometry for annexin V-FITC and propidium iodide (PI) staining. Plots are representative of three independent experiments and the percentages of annexin V positive cells are quantified. (d) MDA-MB-231 cells viability in presence of increasing amounts of DAHP. Nonlinear fitting showed the corresponding IC50. (e) MDA-MB-231 cells and (f) A2780 cells were treated with control, 5 mM DAHP, 10 *μ*M niraparib, or a combination for 72 hours.

The abovementioned results suggested that the PARP inhibitor induced GCH1 expression, which facilitated HRR. However, the restoration of HRR was demonstrated to contribute to the resistance to PARP inhibitor [30–35]. Therefore, we postulated that GCH1 might be closely related to PARP inhibitors' responses and attempted to engage in better inquiry into whether the suppression of GCH1 could boost the efficacy of PARP inhibitors. Small-interfering RNAs (siRNAs) were applied to transiently silence GCH1 in the MDA-MB-231 tumor cell line. The efficacy of knock-down was confirmed by qRT-PCR, and two siRNAs were selected for subsequent experiments (Figure 6(a)). The apoptosis of tumor cells was not significantly changed upon GCH1 silencing (Figure 6(b)). However, the administration of niraparib induced an almost double anticancer effect in siGCH1-transduced cells compared to the control (Figure 6(c)), indicating that inhibition of GCH1 could potentiate PARP inhibitor therapy in cancer. DAHP was reported to uniquely inhibit GCH1 enzymatic activity [36]. Consistently, we observed that DAHP could induce the loss of cell viability with an IC50 ranging from 10 to 15 mM (Figure 6(d)). To further substantiate the hypothesis of a potential synergistic lethal effect between DAHP and PARP inhibitors, the MDA-MB-231 tumor cells were exposed to control, DAHP, niraparib, or a combination. As shown in Figure 6(e) and Supplementary Figure 4a, DAHP or niraparib alone could kill the tumor cells to some extent, but the combination administration gained a significantly better anticancer effect. We obtained similar results using ovarian cancer cell line A2780 (Figure 6(f) and Supplementary Figure 4(b)). The colony formation assay also indicated that niraparib combined with DAHP remarkably suppressed the colony formation capacity of MDA-MB-231 and A2780 cells (Supplementary Figure 4(c)). Collectively, these results suggested that GCH1 attenuated the anticancer activity of niraparib and that inhibiting GCH1 offered a synergistic benefit to PARP inhibitor treatment in vitro.

### 3.6. Inhibition of GCH1 Potentiates the Efficacy of PARP Inhibitor Therapy in Vivo

**Figure 7:**
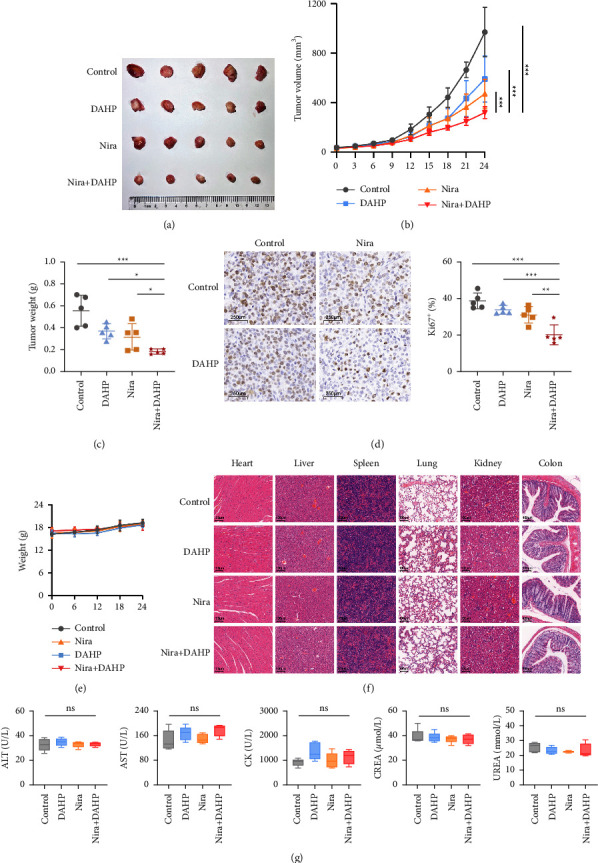
GCH1 inhibitor synergized with niraparib in reducing tumor burden without serious side-effects. (a) Representative images of tumors after niraparib and/or GCH1 inhibitor DAHP treatment in the PDX model. (b) Mean tumor volume of NSG mice subcutaneously implanted with PDX tumors. Tumor-bearing mice were dosed orally with vehicle or niraparib alone or in combination with DAHP. (c) Quantification of tumor weight from tumors described in (b). (d) Comparison of Ki67 expression between the four groups. (e) Quantification of mouse body weights. (f) Representative photos of HE staining of heart, liver, spleen, lung, kidney, and colon after niraparib and/or DAHP treatment. (g) Levels of ALT, AST, CK, CREA, and UREA in serum of the tumor-bearing mice.

On the basis of the benefit achieved by niraparib and the inhibition of GCH1 in vitro, we sought to explore the efficacy using the ovarian cancer PDX model in vivo. Tumors established in NOG mice previously were treated with niraparib and/or GCH1 inhibitor DAHP. Administration of niraparib or DAHP resulted in regression of tumors to a certain extent, whereas combinational therapy significantly reduced tumor volume and tumor weight compared to the monotherapy or vehicle-treated group (Figures 7(a)–7(c)). Ki67 immunohistochemical staining showed a decreased percentage of proliferating (Ki67^+^) tumor cells after the administration of niraparib and DAHP (Figure 7(d)), suggesting that the combination of GCH1 inhibitors and niraparib potently reined back tumor growth. Meanwhile, limited toxic side effects were observed, as evidenced by the lack of decrease in mouse weight (Figure 7(e)), no pathological change of major organs (Figure 7(f)), and no other abnormal biochemical indexes (Figure 7(g)). Taken together, GCH1 inhibitor could augment the sensitivity of tumor cells to PARP inhibitor in vivo.

## 4. Discussion

PARP inhibitors have been approved by the FDA as maintenance therapy for ovarian cancer and breast cancer, and the major obstacle to their clinical application is the inevitable occurrence of acquired drug resistance. An improved understanding of the biological changes of PARP inhibitor-treated tumors would facilitate the design of rational treatment strategies as powerful tools against tumor, postponing the onset of resistance and delaying disease progression. In this context, we screened differentially expressed metabolic enzyme and transporter genes between the niraparib-treated group and the control group and observed significant upregulation of GCH1 expression. Our data demonstrate an increase in GCH1 induced by niraparib at both mRNA and protein levels in cancer cell lines, which was further validated using PDX-derived tissue sections. Moreover, JAK-STAT signaling mediated the upregulation of GCH1 in response to PARP inhibitors. Finally, the relationship of GCH1 to homologous recombination repair was revealed, and we proved the superiority of the combination of PARP inhibitors and GCH1 inhibition in tumor killing in vitro and in vivo. In summary, GCH1 may serve as a promising target to sensitize tumor cells to PARP inhibitors and enhance the antitumor efficacy.

GCH1 is the major rate-limiting enzyme for BH4, an essential cofactor for the production of NOSs [37]. The GCH1/BH4 metabolic pathway participates in the generation of reactive oxygen species and is important in many abnormal disease statuses including diabetes, hypertension, and Parkinson's disease [38–42]. GCH1 protects cells from ferroptosis through the synthesis of BH4/BH2 leading to lipid remodeling in a GPX4-independent manner [43]. The inhibition of GCH1 was proved to be an effective way to manage cancer pain [44]. However, research on the expression and biological functions of GCH1 in cancer is still limited.

Thus, we first evaluated the expression level of GCH1 and found that GCH1 was significantly elevated in breast invasive carcinoma and ovarian cancer. Consistent with our results, GCH1 was also upregulated in glioblastoma [24], indicating its potential hub role in tumorigenesis. We also explored clinical implications of GCH1 and found that higher GCH1 expression was proved to be correlated with several clinicopathological traits.

Despite its clinical value, data on the potential functions and regulating mechanisms involving GCH1 in tumors remain limited. A previous study has reported cytokine-stimulated induction of GCH1 in endothelial cells that TNF-*α* could upregulate GCH1 expression via the activation of IKK/NF-*ĸ*B pathway, while IFN-*γ* had a similar effect attributed to the JAK2/STAT pathway [45]. In this setting, functional annotation of GCH1-associated DEGs was performed. The JAK-STAT signaling pathway, especially transcription factor STAT1, was verified to be positively correlated with GCH1-high expression, highlighting the underlying role of STAT1 in the regulation of GCH1 in breast and ovarian cancers. Further, we observed that administration of niraparib boosted GCH1 expression, which was reversed by inhibition of the JAK-STAT signaling. In spite of the fact that suppression of the JAK-STAT pathway led to a significant decrease of GCH1 expression in MDA-MB-231 cells, it was not evident in A2780 and HCC38 cell lines. This may be due to the inconsistencies in the basal activation levels of the JAK-STAT pathway in different cell lines. Based on the above results, we concluded that niraparib might elevate GCH1 expression via the activation of JAK-STAT pathway.

In the past decades, there has been a great increase in the number of preclinical studies focusing on approaches that could strengthen the PARP inhibitors' cytotoxicity. The loss of the PAR-dependent nucleosome sliding enzyme ALC1/CHD1L conferred up to 250-fold PARP inhibitor hypersensitivity on HR-deficient tumor cells [46]. High expression of CXorf67, a DNA damage response protein that competes with BRCA2 for PALB2 interaction to impair the HR pathway, could increase sensitivity to PARP inhibitors in PFA ependymomas [47]. The autophagy inhibitor chloroquine triggered the deleterious NHEJ DNA repair process and synergistically augmented the anticancer effect of talazoparib in HR-proficient breast cancer [48]. Besides, in HR-proficient ovarian cancer, EZH2 inhibition could promote sensitivity to PARP inhibitors via upregulation of MAD2L2 in a CARM1-dependent manner [49]. Coincidentally, our results revealed that GCH1, which was remarkably upregulated upon niraparib treatment, might be involved in HRR, leading to the resistance to PARP inhibitors. These data provided proof of principle that the synergistic combination of GCH1 suppression and PARP inhibitors was superior, and the superiority was further demonstrated in vitro. Additionally, we have demonstrated in vivo using the PDX model that targeting GCH1 could potentiate the sensitivity of tumor cells to PARP inhibitors and markedly inhibit tumor growth without producing excessive toxic side effects. It is plausible to speculate that certain combinational strategies might provide breast and ovarian cancer patients with more benefits and improve their prognosis. Although we propose a rational therapeutic strategy to render niraparib more effective in killing tumor cells that can be further tested in the clinic, whether GCH1 could be a biomarker for sensitivity to PARP inhibitor treatment still warrants further investigations.

In spite of the merits of the current study, limitations should be addressed. First, part of the data included in the current study on assessing the biological functions of GCH1 was derived from the public TCGA database. Cohorts and studies from other centers are required to validate the findings. Second, the synergistic effects in vivo of GCH1 inhibitors with PARP inhibitors in breast cancer require more detailed exploration due to the limitations of the PDX model.

## 5. Conclusions

In conclusion, we clarified that niraparib induced the upregulation of GCH1, which was associated with sensitivity to PARP inhibitors in ovarian cancer and breast cancer for the first time. This study provides new perspectives for enhancing the potency of PARP inhibitors and offers novel strategies for clinical management of breast and ovarian cancer patients.

## Data Availability

The datasets generated during and/or analyzed during the current study are available from the corresponding author upon reasonable request.

## Supplementary Materials

Supplementary Materials Supplementary Figure 1. Expression profile and prognostic value of SPHK1 in breast and ovarian cancers. (A-B) SPHK1 expression levels in tumor tissues were comparable to normal tissues. n.s: Nonsignificant. Supplementary Figure 2. Top 50 DEGs negatively associated with GCH1 in BRCA and OV patients. ^*∗∗∗*^*P* <  0.001. Supplementary Figure 3. Association of genes highly related to the homologous recombination repair (HRR) pathway and GCH1 expression level in ovarian cancer. Supplementary Figure 4. Pharmacological inhibition of GCH1 enhanced antitumor effects of PARP inhibitor. (A) MDA-MB-231 cells were treated with control, 5 mM DAHP, 10 *μ*M niraparib, or a combination for 24 or 48 hours. (B) The percentage of apoptotic A2780 cells was determined by flow cytometry after treatment with control, 5 mM DAHP, 10 *μ*M niraparib, or a combination for 24 or 48 hours. (C) Colony-formation ability of MDA-MB-231 and A2780 cells was determined upon control, 5 mM DAHP, 10 *μ*M niraparib, or a combination treatment. Representative data and a statistical diagram are shown. Data are presented as the mean ± SD. All experiments were performed at least three times. Statistical significance was determined by one-way ANOVA. ^*∗*^*P* <  0.05, ^*∗∗*^*P*  <  0.01, and ^*∗∗∗*^*P* <  0.001. n.s. Nonsignificant. Supplementary Table 1. DEGs upon niraparib treatment. Supplementary Table 2. The association between GCH1 expression and clinicopathological variables in breast cancer. Supplementary Table 3. The association between GCH1 expression and clinicopathological variables in ovarian cancer. Supplementary Table 4. Logistic regression analysis of association between clinicopathological characteristics and GCH1 expression in breast cancer patients. Supplementary Table 5. Logistic regression analysis of association between clinicopathological characteristics and GCH1 expression in ovarian cancer patients.

## References

[1] Siegel R. L., Miller K. D., Fuchs H. E., Jemal A. (2022). Cancer statistics, 2022. *CA: A Cancer Journal for Clinicians*.

[2] Harbeck N., Penault-Llorca F., Cortes J. (2019). Breast cancer. *Nature Reviews Disease Primers*.

[3] Kuroki L., Guntupalli S. R. (2020). Treatment of epithelial ovarian cancer. *BMJ*.

[4] Cohen M. S., Chang P. (2018). Insights into the biogenesis, function, and regulation of ADP-ribosylation. *Nature Chemical Biology*.

[5] Palazzo L., Ahel I. (2018). PARPs in genome stability and signal transduction: implications for cancer therapy. *Biochemical Society Transactions*.

[6] Gonzalez Martin A., Pothuri B., Vergote I. (2019). Niraparib in patients with newly diagnosed advanced ovarian cancer. *New England Journal of Medicine*.

[7] Robson M., Im S. A., Senkus E. (2017). Olaparib for metastatic breast cancer in patients with a germline BRCA mutation. *New England Journal of Medicine*.

[8] de Bono J., Mateo J., Fizazi K. (2020). Olaparib for metastatic castration-resistant prostate cancer. *New England Journal of Medicine*.

[9] Golan T., Hammel P., Reni M. (2019). Maintenance olaparib for germline BRCA-mutated metastatic pancreatic cancer. *New England Journal of Medicine*.

[10] Dias M. P., Moser S. C., Ganesan S., Jonkers J. (2021). Understanding and overcoming resistance to PARP inhibitors in cancer therapy. *Nature Reviews Clinical Oncology*.

[11] Ibrahim Y. H., Garcia-Garcia C., Serra V. (2012). PI3K inhibition impairs BRCA1/2 expression and sensitizes BRCA-proficient triple-negative breast cancer to PARP inhibition. *Cancer Discovery*.

[12] Juvekar A., Burga L. N., Hu H. (2012). Combining a PI3K inhibitor with a PARP inhibitor provides an effective therapy for BRCA1-related breast cancer. *Cancer Discovery*.

[13] Yap T. A., Kristeleit R., Michalarea V. (2020). Phase I trial of the PARP inhibitor olaparib and AKT inhibitor capivasertib in patients with BRCA1/2- and non-BRCA1/2-mutant cancers. *Cancer Discovery*.

[14] Pai Bellare G., Sankar Patro B. (2022). Resveratrol sensitizes breast cancer to PARP inhibitor, talazoparib through dual inhibition of AKT and autophagy flux. *Biochemical Pharmacology*.

[15] Bajrami I., Kigozi A., Van Weverwijk A. (2012). Synthetic lethality of PARP and NAMPT inhibition in triple-negative breast cancer cells. *EMBO Molecular Medicine*.

[16] Domchek S. M., Postel-Vinay S., Im S. A. (2020). Olaparib and durvalumab in patients with germline BRCA-mutated metastatic breast cancer (MEDIOLA): an open-label, multicentre, phase 1/2, basket study. *The Lancet Oncology*.

[17] Konstantinopoulos P. A., Waggoner S., Vidal G. A. (2019). Single-arm phases 1 and 2 trial of niraparib in combination with pembrolizumab in patients with recurrent platinum-resistant ovarian carcinoma. *JAMA Oncology*.

[18] Jiao S., Xia W., Yamaguchi H. (2017). PARP inhibitor upregulates PD-L1 expression and enhances cancer-associated immunosuppression. *Clinical Cancer Research*.

[19] Shen J., Zhao W., Ju Z. (2019). PARPi triggers the STING-dependent immune response and enhances the therapeutic efficacy of immune checkpoint blockade independent of BRCAness. *Cancer Research*.

[20] Qu J., Sun W., Zhong J. (2017). Phosphoglycerate mutase 1 regulates dNTP pool and promotes homologous recombination repair in cancer cells. *Journal of Cell Biology*.

[21] Alp N. J., Mussa S., Khoo J. (2003). Tetrahydrobiopterin-dependent preservation of nitric oxide-mediated endothelial function in diabetes by targeted transgenic GTP-cyclohydrolase I overexpression. *Journal of Clinical Investigation*.

[22] Cai S., Khoo J., Channon K. M. (2005). Augmented BH4 by gene transfer restores nitric oxide synthase function in hyperglycemic human endothelial cells. *Cardiovascular Research*.

[23] Pickert G., Lim H. Y., Weigert A. (2013). Inhibition of GTP cyclohydrolase attenuates tumor growth by reducing angiogenesis and M2-like polarization of tumor associated macrophages. *International Journal of Cancer*.

[24] Tran A. N., Walker K., Harrison D. G. (2018). Reactive species balance via GTP cyclohydrolase I regulates glioblastoma growth and tumor initiating cell maintenance. *Neuro-Oncology*.

[25] Possemato R., Marks K. M., Shaul Y. D. (2011). Functional genomics reveal that the serine synthesis pathway is essential in breast cancer. *Nature*.

[26] Love M. I., Huber W., Anders S. (2014). Moderated estimation of fold change and dispersion for RNA-seq data with DESeq2. *Genome Biology*.

[27] Wickham H. (2009). *ggplot2: Elegant Graphics for Data Analysis*.

[28] Blighe K., Rana S., Lewis M. (2018). *EnhancedVolcano: Publication-Ready Volcano Plots With Enhanced Colouring and Labeling*.

[29] Ding L., Chen X., Xu X. (2019). PARP1 suppresses the transcription of PD-L1 by poly(ADP-ribosyl)ating STAT3. *Cancer Immunology Research*.

[30] Barber L. J., Sandhu S., Chen L. (2013). Secondary mutations in BRCA2 associated with clinical resistance to a PARP inhibitor. *The Journal of Pathology*.

[31] Bouwman P., Aly A., Escandell J. M. (2010). 53BP1 loss rescues BRCA1 deficiency and is associated with triple-negative and BRCA-mutated breast cancers. *Nature Structural & Molecular Biology*.

[32] Edwards S. L., Brough R., Lord C. J. (2008). Resistance to therapy caused by intragenic deletion in BRCA2. *Nature*.

[33] Kondrashova O., Nguyen M., Shield-Artin K. (2017). Secondary somatic mutations restoring RAD51C and RAD51D associated with acquired resistance to the PARP inhibitor rucaparib in high-grade ovarian carcinoma. *Cancer Discovery*.

[34] Pettitt S. J., Frankum J. R., Punta M. (2020). Clinical BRCA1/2 reversion analysis identifies hotspot mutations and predicted neoantigens associated with therapy resistance. *Cancer Discovery*.

[35] Xu G., Chapman J. R., Brandsma I. (2015). REV7 counteracts DNA double-strand break resection and affects PARP inhibition. *Nature*.

[36] Xie L., Smith J. A., Gross S. S. (1998). GTP cyclohydrolase I inhibition by the prototypic inhibitor 2,4-dh. *Journal of Biological Chemistry*.

[37] Werner E. R., Blau N., Thony B. (2011). Tetrahydrobiopterin: biochemistry and pathophysiology. *Biochemical Journal*.

[38] Ge Z. D., Ionova I. A., Vladic N. (2011). Cardiac-specific overexpression of GTP cyclohydrolase 1 restores ischaemic preconditioning during hyperglycaemia. *Cardiovascular Research*.

[39] Kidokoro K., Satoh M., Channon K. M., Yada T., Sasaki T., Kashihara N. (2013). Maintenance of endothelial guanosine triphosphate cyclohydrolase I ameliorates diabetic nephropathy. *Journal of the American Society of Nephrology*.

[40] Nagatsu T., Nakashima A., Ichinose H., Kobayashi K. (2019). Human tyrosine hydroxylase in Parkinson’s disease and in related disorders. *Journal of Neural Transmission*.

[41] Stroes E., Kastelein J., Cosentino F. (1997). Tetrahydrobiopterin restores endothelial function in hypercholesterolemia. *Journal of Clinical Investigation*.

[42] Tie L., Chen L. Y., Chen D. D., Xie H. H., Channon K. M., Chen A. F. (2014). GTP cyclohydrolase I prevents diabetic-impaired endothelial progenitor cells and wound healing by suppressing oxidative stress/thrombospondin-1. *American Journal of Physiology - Endocrinology And Metabolism*.

[43] Kraft V. A. N., Bezjian C. T., Pfeiffer S. (2020). GTP cyclohydrolase 1/tetrahydrobiopterin counteract ferroptosis through lipid remodeling. *ACS Central Science*.

[44] Pickert G., Myrczek T., Ruckert S. (2012). Inhibition of GTP cyclohydrolase reduces cancer pain in mice and enhances analgesic effects of morphine. *Journal of Molecular Medicine (Berlin)*.

[45] Huang A., Zhang Y.-Y., Chen K., Hatakeyama K., Keaney J. F. (2005). Cytokine-stimulated GTP cyclohydrolase I expression in endothelial cells requires coordinated activation of nuclear factor-*κ*b and stat1/stat3. *Circulation Research*.

[46] Verma P., Zhou Y., Cao Z. (2021). ALC1 links chromatin accessibility to PARP inhibitor response in homologous recombination-deficient cells. *Nature Cell Biology*.

[47] Han J., Yu M., Bai Y. (2020). Elevated CXorf67 expression in PFA ependymomas suppresses DNA repair and sensitizes to PARP inhibitors. *Cancer Cell*.

[48] Pai Bellare G., Saha B., Patro B. S. (2021). Targeting autophagy reverses de novo resistance in homologous recombination repair proficient breast cancers to PARP inhibition. *British Journal of Cancer*.

[49] Karakashev S., Fukumoto T., Zhao B. (2020). EZH2 inhibition sensitizes CARM1-high, homologous recombination proficient ovarian cancers to PARP inhibition. *Cancer Cell*.

