# Supplementary material for: Suppression of GCH1 Sensitizes Ovarian Cancer and Breast Cancer to PARP Inhibitor
Source: J Oncol. 2023 Feb 6;2023:1453739. doi: 10.1155/2023/1453739 (PMC9925261; doi:10.1155/2023/1453739)
Supplement: Supplementary Materials — Supplementary Figure 1. Expression profile and prognostic value of SPHK1 in breast and ovarian cancers. (A-B) SPHK1 expression levels in tumor tissues were comparable to normal tissues. n.s: Nonsignificant. Supplementary Figure 2. Top 50 DEGs negatively associated with GCH1 in BRCA and OV patients. ∗∗∗P < 0.001. Supplementary Figure 3. Association of genes highly related to the homologous recombination repair (HRR) pathway and GCH1 expression level in ovarian cancer. Supplementary Figure 4. Pharmacological inhibition of GCH1 enhanced antitumor effects of PARP inhibitor. (A) MDA-MB-231 cells were treated with control, 5 mM DAHP, 10 μM niraparib, or a combination for 24 or 48 hours. (B) The percentage of apoptotic A2780 cells was determined by flow cytometry after treatment with control, 5 mM DAHP, 10 μM niraparib, or a combination for 24 or 48 hours. (C) Colony-formation ability of MDA-MB-231 and A2780 cells was determined upon control, 5 mM DAHP, 10 μM niraparib, or a combination treatment. Representative data and a statistical diagram are shown. Data are presented as the mean ± SD. All experiments were performed at least three times. Statistical significance was determined by one-way ANOVA. ∗P < 0.05, ∗∗P < 0.01, and ∗∗∗P < 0.001. n.s. Nonsignificant. Supplementary Table 1. DEGs upon niraparib treatment. Supplementary Table 2. The association between GCH1 expression and clinicopathological variables in breast cancer. Supplementary Table 3. The association between GCH1 expression and clinicopathological variables in ovarian cancer. Supplementary Table 4. Logistic regression analysis of association between clinicopathological characteristics and GCH1 expression in breast cancer patients. Supplementary Table 5. Logistic regression analysis of association between clinicopathological characteristics and GCH1 expression in ovarian cancer patients. [file 1453739.f1.zip › Supplementary Table 2.docx]

**Supplementary Table 2**. The association between GCH1 expression and clinicopathological variables in breast cancer.

| **Characteristic** | **levels** | **Low expression of GCH1** | **High expression of GCH1** | **p** |
| --- | --- | --- | --- | --- |
| N |  | 541 | 542 |  |
| T stage (%) | T1 | 140 (13%) | 137 (12.7%) | **0.002** |
|  | T2 | 299 (27.7%) | 330 (30.6%) |  |
|  | T3 | 88 (8.1%) | 51 (4.7%) |  |
|  | T4 | 12 (1.1%) | 23 (2.1%) |  |
| N stage (%) | N0 | 260 (24.4%) | 254 (23.9%) | 0.069 |
|  | N1 | 183 (17.2%) | 175 (16.4%) |  |
|  | N2 | 46 (4.3%) | 70 (6.6%) |  |
|  | N3 | 44 (4.1%) | 32 (3%) |  |
| M stage (%) | M0 | 433 (47%) | 469 (50.9%) | 0.968 |
|  | M1 | 9 (1%) | 11 (1.2%) |  |
| Pathologic stage (%) | Stage I | 90 (8.5%) | 91 (8.6%) | 0.972 |
|  | Stage II | 313 (29.5%) | 306 (28.9%) |  |
|  | Stage III | 118 (11.1%) | 124 (11.7%) |  |
|  | Stage IV | 9 (0.8%) | 9 (0.8%) |  |
| Race (%) | Asian | 22 (2.2%) | 38 (3.8%) | 0.088 |
|  | Black or African American | 95 (9.6%) | 86 (8.7%) |  |
|  | White | 382 (38.4%) | 371 (37.3%) |  |
| Age (%) | <=60 | 297 (27.4%) | 304 (28.1%) | 0.739 |
|  | >60 | 244 (22.5%) | 238 (22%) |  |
| Histological type (%) | Infiltrating Ductal Carcinoma | 344 (35.2%) | 428 (43.8%) | **< 0.001** |
|  | Infiltrating Lobular Carcinoma | 143 (14.6%) | 62 (6.3%) |  |
| PR status (%) | Negative | 149 (14.4%) | 193 (18.7%) | **0.001** |
|  | Indeterminate | 2 (0.2%) | 2 (0.2%) |  |
|  | Positive | 378 (36.6%) | 310 (30%) |  |
| ER status (%) | Negative | 92 (8.9%) | 148 (14.3%) | **< 0.001** |
|  | Indeterminate | 1 (0.1%) | 1 (0.1%) |  |
|  | Positive | 437 (42.2%) | 356 (34.4%) |  |
| HER2 status (%) | Negative | 297 (40.9%) | 261 (35.9%) | **0.004** |
|  | Indeterminate | 6 (0.8%) | 6 (0.8%) |  |
|  | Positive | 60 (8.3%) | 97 (13.3%) |  |
| PAM50 (%) | Normal | 18 (1.7%) | 22 (2%) | **< 0.001** |
|  | LumA | 354 (32.7%) | 208 (19.2%) |  |
|  | LumB | 64 (5.9%) | 140 (12.9%) |  |
|  | Her2 | 18 (1.7%) | 64 (5.9%) |  |
|  | Basal | 87 (8%) | 108 (10%) |  |
| Menopause status (%) | Pre | 108 (11.1%) | 121 (12.4%) | 0.483 |
|  | Peri | 18 (1.9%) | 22 (2.3%) |  |
|  | Post | 359 (36.9%) | 344 (35.4%) |  |
| Anatomic neoplasm subdivisions (%) | Left | 285 (26.3%) | 278 (25.7%) | 0.692 |
|  | Right | 256 (23.6%) | 264 (24.4%) |  |
| Radiation therapy (%) | No | 220 (22.3%) | 214 (21.7%) | 0.857 |
|  | Yes | 276 (28%) | 277 (28.1%) |  |
| OS event (%) | Alive | 472 (43.6%) | 459 (42.4%) | 0.261 |
|  | Dead | 69 (6.4%) | 83 (7.7%) |  |
| DSS event (%) | Alive | 497 (46.8%) | 481 (45.2%) | 0.240 |
|  | Dead | 37 (3.5%) | 48 (4.5%) |  |
| PFI event (%) | Alive | 467 (43.1%) | 469 (43.3%) | 0.990 |
|  | Dead | 74 (6.8%) | 73 (6.7%) |  |
| Age, median (IQR) |  | 59 (49, 67) | 58 (48, 67) | 0.709 |
